# Detection and Removal of Aristolochic Acid in Natural Plants, Pharmaceuticals, and Environmental and Biological Samples: A Review

**DOI:** 10.3390/molecules29010081

**Published:** 2023-12-22

**Authors:** Changhong Wang, Yunchao Liu, Jintai Han, Wenying Li, Jing Sun, Yinan Wang

**Affiliations:** 1 School of Environmental Science and Engineering, Qilu University of Technology (Shandong Academy of Sciences), Jinan 250353, China; wangch001@126.com (C.W.); 15066722550@163.com (Y.L.); hanjt628@163.com (J.H.); 2 College of Geography and Environment, Shandong Normal University, Jinan 250000, China; 15610107112@163.com; 3 Key Laboratory of Fine Chemicals in Universities of Shandong, Jinan Engineering Laboratory for Multi-Scale Functional Materials, School of Chemistry and Chemical Engineering, Qilu University of Technology (Shandong Academy of Sciences), Jinan 250353, China

**Keywords:** aristolochic acids, aristolochic acid nephropathy, Balkan endemic nephropathy, remove, detection

## Abstract

Aristolochic acids (AAs) are a toxic substance present in certain natural plants. Direct human exposure to these plants containing AAs leads to a severe and irreversible condition known as aristolochic acid nephropathy (AAN). Additionally, AAs accumulation in the food chain through environmental mediators can trigger Balkan endemic nephropathy (BEN), an environmental variant of AAN. This paper presents a concise overview of the oncogenic pathways associated with AAs and explores the various routes of environmental exposure to AAs. The detection and removal of AAs in natural plants, drugs, and environmental and biological samples were classified and summarized, and the advantages and disadvantages of the various methods were analyzed. It is hoped that this review can provide effective insights into the detection and removal of AAs in the future.

## 1. Introduction

The increasing global concern for health has led to a rise in the use of natural medicines, with nearly 80% of the world’s population now relying on them for healthcare, as reported by the World Health Organization (WHO) [1]. However, the safety of these medications is often overlooked. Some herbal medicines may contain harmful components that can be detrimental to the kidneys, which are more susceptible to damage from drugs and toxins than other organs [2]. One such harmful component is aristolochic acids (AAs), a group of necrophilic carboxylic acids, which has shed light on the dangers associated with plants from the Aristolochia genus. The Aristolochiaceae family, consisting of seven genera and 400 species, is widely distributed in the Mediterranean, Africa, and Asia [3,4]. More than a century ago, Aristolochia plants were extensively used in traditional medicine and pharmacology, including applications as anti-inflammatory drugs and analgesics. However, due to the increasing occurrence of malignant incidents associated with these plants, their toxicity is gradually being confirmed.

The link between aristolochic acid nephropathy and natural products originated from events in Belgium during the 1990s, when a doctor mistakenly included *Fang Chi Root* in diet pills, resulting in nine young women being diagnosed with rapidly progressive fibrosing interstitial nephritis [5]. Since then, nephritis caused by herbs has been referred to as Chinese herbs nephropathy (CHN) [6]. In 1996, Schmeiser et al. [7] tested kidney tissue from affected patients and isolated aristolochic acid-DNA adducts. Subsequently, Wolf et al. [8] associated the increasing number of renal impairment cases and progressively complex symptoms with the term “aristolochic acid nephropathy” (AAN). AAN primarily manifests as progressive interstitial renal fibrosis [9], which can progress to end-stage renal disease and be followed by upper urinary tract urothelial carcinoma and bladder urothelial cancer [10]. The carcinogenicity of AAs is attributed to their irreversible nature, and appropriate measures should be taken to control exposure to these compounds to prevent cancer. In recent years, several effective countermeasures have been implemented worldwide to address the damage caused by AAs. In 2000, the European Medicines Agency reminded EU member states to avoid the use of Aristolochia species or as a result of confusion with other botanical plants [11]. According to this recommendation, most EU member states’ authorities have taken risk management actions. In 2001, the WHO issued a warning about aristolochic acid. In 2002, the International Agency for Research on Cancer (IARC) listed AA-containing plants as group I carcinogens for the first time [12]. Subsequently, such plants appeared on banned lists in several countries. In 2012, the IARC classified AAs as group I carcinogens for human cancer [13]. So far, most countries have banned medicines that are suspected to contain or actually contain AAs.

Apart from kidney disease, AAs may also affect other organs. Ng et al. [14] were the first to propose the idea that distinctive mutations caused by AAs may induce hepatocellular carcinoma. They tested and compared whole exons from hepatocellular carcinoma cells but did not have absolute evidence of the relationship between aristolochic acid exposure and liver cancer. Lu et al. [15] concluded that one of the components of AAs, AAI, can cause liver cancer in a dose-dependent manner based on experiments in a mouse model. Additionally, Shi et al. investigated the effects of sex and age on the carcinogenic capacity of AAs and found that female patients had a lower rate of renal failure due to the inhibitory effect of 17-estradiol on AAN. When exposure was prolonged, AAI caused more severe liver damage in newborn mice than in adult mice [16]. AAs not only have a high mortality rate but also accumulate in the environment and indirectly enter the human body through the food chain, leading to a range of diseases. One example of the environmental transmission of AAN is the emergence of Balkan endemic nephropathy (BEN), predominantly occurring in rural areas along the tributaries of the Danube in Balkan countries [17]. The conclusion is that AAs are difficult to remove in soil and are strongly retained by the soil matrix [18]. Investigations into the cause of the disease revealed that the long-term consumption of wheat flour contaminated with AAs could be responsible [19,20], thus establishing the link between AAs and the food chain [21]. AAs have irreversible effects on human health, and the etiology of Balkan endemic nephropathy can be attributed to environmental exposure to AAs. The exposure channels through environmental media include soil, atmosphere, and water. It is important to note that natural plants containing AAs are not limited to the Balkans; wherever plants with similar natural ingredients grow, there is a risk of adverse effects on human health through the food chain [22]. All of these relevant attenuation and detoxification studies have been carried out after AAs have entered the human body. However, to significantly reduce the number of AAN cases, it is crucial to minimize exposure to AAs. In addition to improving the identification of analogs, it is essential to find ways to eliminate AAs from Chinese medicine and contaminated environmental media, thereby reducing the probability of AAs accumulation through the food chain.

This article briefly reviews the pathogenicity of AAs and possible transmission routes in the environment. This review focuses on the detection and removal of AAs in natural plants, drugs, environment, and biological samples and analyzes the advantages and disadvantages of each method. This should further stimulate the creativity of researchers in detecting and removing AAs and promote the development of this direction. It is hoped that this paper can play a role in sorting out and looking forward to the identification and detection of AAs.

## 2. AAs Can Cause Cancer and Can Be Exposed to the Environment

This section highlights the irreversible pathogenicity of AAs and the urgent need to address their presence in the environment. It briefly discusses the carcinogenic pathways of AAs and their transportation through various environmental media.

### 2.1. How Do AAs Cause Cancer?

**Figure 1:**
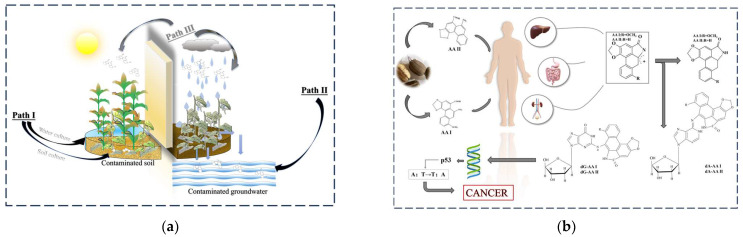
(**a**) Exposure pathways of AAs in the environment (AAI and AAII as examples). (**b**) Carcinogenic pathways of AAI and AAII.

In 1969, AAs were linked to the etiology of BEN when the regional concentration of kidney cancer patients was observed [23]. It has been demonstrated that BEN is caused by a genotoxic mutagen produced by AAs after metabolism in the human body [24]. To elucidate the pathway further, researchers utilized ^32^P-labeled DNA adducts to trace the metabolic pathways of AAs in humans [25]. The two most common AAs, AAI and AAII, were chosen as representative substances for this study. In summary, as shown in Figure 1a, AAs enter the body and initially generate N-hydroxyaristolactams (AL-NOHs) through nitroreduction reactions [10]. These AL-NOHs form covalent DNA adducts by binding preferentially to the exocyclic amino acid group of purine nucleotides, creating a cyclic N-acylnitrenium ion with a delocalized positive charge [26]. Intermediate products, including 7-(deoxyguanosin-N^2^-yl)-aristolactam I (dG-AAI) and 7-(deoxyadenosine-N^6^-yl)-aristolactam I (dA-AAI), are formed through the reaction of AAI with xanthine oxidase [27]. AAI and AAII undergo demethoxylation to produce 7-(deoxyadenosin-N^6^-yl)-aristolactam II (dA-AAII) and 7-(deoxyguanosin-N^2^-yl)-aristolactam II (dG-AAII), respectively. AAII reacts with the deoxyguanosine 3′-monophosphate of DNA to form dG-AAII. Notably, the dA adduct exhibits significantly higher mutagenic potential than the dG adduct [28]. These formed adducts induce mutations in the tumor suppressor gene p53, with 89% of the mutations involving the A:T pair and resulting in A:T → T:A translocations. Subsequent mutations lead to the development of cancer [29].

The pathway highlights the significance of DNA adducts in the disease process, prompting further research of the mutagenic and carcinogenic potential of AAs. Arlt et al. [30] conducted a systematic review exploring the genotoxic mechanism underlying AAI and AAII carcinogenicity, focusing on the mutagenic activity of DNA adducts and their combination specificity. Building on this work, Jadot et al. [31] delved deeper into the underlying mechanisms of AAN, particularly vascular events. Additionally, extensive attention has been given to studying the toxicity and mechanisms of AA analogues other than AAI and AAII. In a toxicity study of AAIVa, Xian et al. [32] proposed an additional oncogenic pathway, suggesting that the activation of the typical or atypical transforming growth factor-β pathway could lead to mild renal lesions. This research provides a foundation for subsequent toxicity studies on analogues.

### 2.2. Exposure to AAs in the Environment

BEN is a disease resulting from people’s exposure to AAs through environmental media. Although the ingestion of AAs in the environment is relatively low compared to AAN, it manifests differently clinically. BEN is characterized by a high prevalence and a long latent period [33]. Prolonged exposure to one or more AA-containing environmental factors is the primary cause of the disease. These factors enter the food chain through environmental media and undergo bioaccumulation, leading to human disease [34].

To explore the etiology of BEN, it was later confirmed that naturally occurring plant roots can absorb AAs from nutrient solutions, thereby establishing the possibility of AAs uptake by food crops in the food chain [35,36]. As depicted in Figure 1b, Chan et al. [37] examined crop and soil samples collected in Serbia, specifically maize and wheat, and demonstrated that both food crops and soils in the Balkans were contaminated with AAs. It was concluded that the edible parts of crops grown in contaminated soils could be one of the pathways for human exposure to AAs (Path I) [38]. AAs exhibit high stability in soil [36]. Soil samples from herb-growing areas contaminated with AAs have been detected [39]. Zhang et al. [40] conducted experiments using AA-contaminated soil to grow three typical vegetables, revealing a positive relationship between the concentration of AAs in the soil and the concentration of AAs within the vegetables. AAs can also accumulate in hydroponically cultured plants [35,41]. Through leaching and infiltration, AAs from decomposed natural plants can reach groundwater, significantly contaminating drinking water in Serbian villages (Path II) [42]. After confirming the two environmental pathways of water and soil, Alexandra T et al. explored soils from BEN and non-BEN areas in comparative experiments, proposing a potential respiratory hypothesis as a new exposure pathway (Path III). The airborne spread of AAs is currently only speculative and lacks confirmation. Regarding the environmental exposure pathways of AAI, Alexandra T et al. [43] conducted a systematic review providing a comprehensive description of these pathways.

## 3. Detection Methods of AAs

At present, the detection of AAs has been applied to natural plants, drugs, and environmental and biological samples. Common detection methods include high-performance liquid chromatography (HPLC), high-performance liquid chromatography tandem mass spectrometry (HPLC-MS), etc. Other detection methods comprise the fluorescence sensor method, near-infrared spectroscopy, biological detection method, and so on.

### 3.1. Common Detection Methods

#### 3.1.1. HPLC

Among the detection methods for detecting AAs, HPLC is the most mature method with high sensitivity, high accuracy, and good reproducibility. The detectors used include an ultraviolet (UV) detector, fluorescence detector (FLD), and electrochemical detector (ECD). Lee et al. [44] used a mobile phase consisting of 0.3% ammonium carbonate/acetonitrile (75:25, *v*/*v*) to detect AAI and AAII from a drug under a UV detector within 20 min. Akira et al. [45] applied for the first time electrochemical reduction to detect AAI and AAII through HPLC-ECD. The AAs without the fluorescence effect were further transformed into luminescent aristolochic acid lactams (ALs) using zinc powder and detected by HPLC-FLD [46,47]. Finally, the content of ALs was deducted from the total amount to obtain the amounts of AAI and AAII. To further improve the selectivity of HPLC-FLD for the detection of AAI and AAII, Wang et al. [48] used the cysteine-induced hydrogenolysis of AAs to replace the nitro group that inhibits fluorescence through hydrogen and reduce interference. Compared with UV and ECD, FLD is the most sensitive and selective detector and is thus used more frequently. Chan et al. [37] used Zn/H^+^ to produce fluorescent nitro reduction products to detect AAs in crops and soils in Serbia. Li et al. [49] applied Zn/H^+^ in denitrification to detect AAs, which were detected fluorescently at excitation and emission wavelengths of 393 and 470 nm, respectively. Preprocessing greatly increases the cost and time of detection. To reduce cost, Chin et al. [50] developed a simple and efficient iron powder packaging reduction column, which can be used for the simultaneous monitoring and quantification of AAs in one run. This column breaks through the original HPLC-FLD and has a certain development prospect.

#### 3.1.2. HPLC-MS

Single-operation HPLC is insufficient for the detection of trace of AAs (µg/kg) in complex matrices. MS plays an important role in disease development, drug treatment, environmental exposure, and other fields [51]. To improve the sensitivity and specificity of the analysis, Chan et al. [52] used liquid chromatography–electrospray tandem mass spectrometry and a quadrupole time-of-flight mass spectrometer to analyze the adducts of AAs via MS-MS. Elvis et al. [53] used the same method to quantify aristolochic acid-RNA adducts in rat urine. Draghia et al. [54] developed an ultra-high-performance liquid chromatography combined with an ion-trap mass spectrometer system (UHPLC-IT-MS) for the detection and quantification of AAI in crops and surrounding soil, which improved the detection sensitivity of analogues. The researchers optimized the detection accuracy by changing the type of mass spectrometer. Table 1 shows the integrated analytical methods, samples, mass spectrometer types, detection modes, components, and scanning modes used in related studies.

Despite the higher selectivity of HPLC-MS compared to HPLC, the equipment is relatively expensive and requires professional operation, and the sensitivity is overly dependent on ionization technology. With the development of HPLC-MS technology, other rapid detection methods have been developed.

### 3.2. Other Detection Methods

#### 3.2.1. Sensors

Sensors have attracted increasing attention in the field of the rapid detection of AAs due to their advantages of fast response, low cost, and simple operation. Fluorescence sensors detect analytes through fluorescence signals; the reaction capability and sensitivity of sensors are optimized by improving the fluorescence probe. Liu et al. [69] prepared a novel fluorescent graft conjugated polymer poly (PPE-OB-PEG) for the detection of AAI. The experimental results showed the great potential of PPE-OB-PEG in the field of the rapid detection of AAI in Chinese patent medicines. Song et al. [70] synthesized a luminescent metal–organic framework (Zn-MOF) probe with excellent fluorescence stability, and it can detect AAs in biological liquids with a low detection limit. Shen et al. [71] used a simple ion-exchange method to synthesize the ionic covalent organic framework TGH^+^ PD: Eu(TTA)_4_ with lanthanide emission characteristics; they also used COFs for the first time as fluorescent probes for the bimodal detection of AAs in the human body and antibiotics in wastewater with high selectivity. Zhu et al. prepared a new type of Eu^3+^-functionalized hydrogen-bonded organic framework (Eu@HOF-BTB, Eu@1) that can simultaneously distinguish rosmarinic and AAs; the sensor exhibited a higher selectivity than the previous one. In addition, the response signal can be enhanced through the addition of small-scale nanomaterials to the chemiluminescence system. Oraby et al. [71] introduced gold nanoparticles with chemical stability and high surface oxidation resistance to a chemiluminescence system for the detection of AAI in medicinal plant materials and commercially available weight-loss products. Although the fluorescence sensor had high sensitivity and selectivity, some fluorescent materials were unstable under light, which resulted in inaccurate detection. The detection of AAs using an external voltage at a high pH has gradually attracted widespread attention. Li et al. [72] reported for the first time the use of an electrochemical method for the rapid analysis of AAI and AAII in medicinal plant samples and with β-cyclodextrin as a modifier. The quantitative experiment was realized via a cyclic voltage test on the traditional ball–beam electrode, which has a lower detection limit and a wider linear range. Wang et al. prepared cyclic voltammetry sensors by modifying glassy carbon electrode materials to improve the sensitivity of detection. MoS_2_@MWCNTs nanocomposites [73] and ordered mesoporous carbon materials [74] were used to detect AAs via cyclic voltammetry and linear sweep voltammetry. Although the sensor has a low detection limit, a high test range, and relatively low cost, it exhibits poor stability during detection and is easily affected by pH and temperature.

#### 3.2.2. Near-Infrared Spectroscopy

Near-infrared spectroscopy is an efficient, nondestructive, and nonpolluting detection method. Based on the vibration absorption of molecular groups in the sample [75], an unknown sample is compared with an established calibration model for qualitative and quantitative analysis. This method requires a small amount of sample and produces no secondary pollutants. Therefore, near-infrared spectroscopy has been widely used in the detection of pollutants in food and the environment. Chen et al. [76] developed an end-to-end one-dimensional convolutional neural network (1D-CNN) model based on near-infrared spectroscopy to distinguish Aas and their analogues; this model can extract characteristic wavelengths from original input data without requiring manual selection. This proposed model also saves manpower and shortens the detection time. However, this method is also susceptible to external influences, and the external environment leads to changes in the absorption peak, which reduces detection accuracy.

#### 3.2.3. Biological Detection Methods

**Figure 2:**
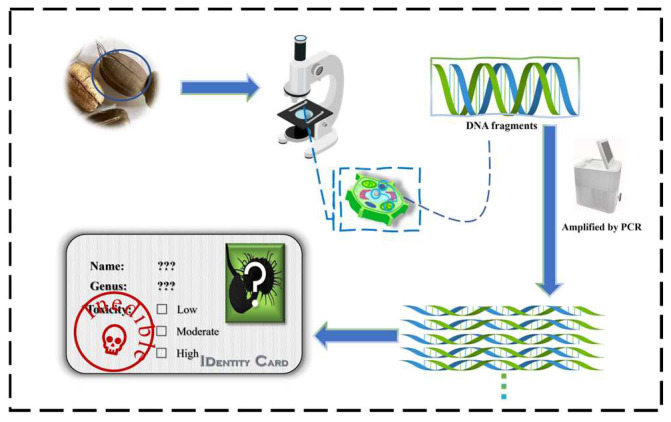
DNA barcode technology route.

Aristolochia plants contain AAs, and the detection of natural plants should take into account factors such as harvest time and geographical differences, so it is difficult to identify plants using chemicals [77]. The use of nucleotide differences between species is a simple and reliable technique for distinguishing target species from other species. The method is used to track the original species by DNA barcode technology and identify Aristolochia plants [78]. DNA barcoding is illustrated in Figure 2. First, a section of the plant’s DNA is extracted, which is then amplified using a PCR amplifier. The product is then purified, and its sequence is determined and quality assessed. This makes each plant its own unique “identity card”. Wu et al. [79] were able to successfully categorize 158 Aristolochia samples and 131 non-Aristolochia alternatives using DNA barcoding techniques based on ITS2 and psbA-trnH sequences in conjunction with BLAST identification and neighbor-joining methods. In addition, breakthroughs in identifying drug species were made by using second-generation (massively parallel sequencing) and third-generation (single molecule sequencing) sequencing technologies, allowing for the traceability analysis of species in multidrug mixtures to be conducted. For example, Xin et al. [80] applied DNA barcoding and single-molecule (real-time, SMRT) sequencing to detect components in Jiuwei Qianghuo Wan and homemade preparations. In order to reduce the cost and simplify the species analysis procedure, CGICA was introduced into the rapid detection of AAs. Kannika et al. [81] developed a polymerase chain reaction combined with a lateral flow immunochromatography assay (PCR-LFA) for the detection of aristolochia plants and related herbal products based on the nucleotide characteristics of the rbcL gene region of aristolochia species. The sensitivity of the lateral flow strip to detect amplicons of genomic DNA amplification was as low as 0.01 ng. At the same time, Chen et al. [82] synthesized two immunochromatographic test strips based on gold nanospheres and gold nanoflowers for the simultaneous rapid detection of aflatoxins and AAI in medicinal and edible materials. Compared with traditional detection methods, biological detection methods do not require expensive equipment and are suitable for on-site and batch detection, but the quantitative results are not as accurate as traditional methods.

## 4. Removal of AAs

The toxicity of AAs limits the clinical applications of drugs containing such components. Therefore, a suitable method for the removal of such components from drugs must be sought. Researchers have conducted extensive research to reduce the toxicity of traditional Chinese medicine containing AAs. The current methods include microbiological, physical adsorption separation, and chemical methods.

### 4.1. Microbiological Methods

Microbes can break down and transform herbal medicines [83]. Microbiological techniques are currently being used to ferment traditional Chinese medicines to obtain active substances [84]. Zikmundov et al. [85] studied antibiotic biotransformation pathways in plants using four fungi and analyzed their mechanisms of transformation. Melo et al. [86] cocultured AAI and AAII with Botrytis cinerea and analyzed their possible biotransformation by the said organism, which laid the foundation for the exploration of the biological effects of microorganisms on AAs. Wang et al. [87] experimentally identified a fungus, Neocosmospora solani, that degrades AAI and screened the candidate genes involved. In addition, the degradation mechanism between the fungus and AAI was revealed, which further provided a new method for the detoxification of traditional Chinese medicine plant materials. Compared with other technologies, microbial remediation technology can effectively avoid secondary pollution problems and has a low cost. However, this technology requires a long repair time and faces certain limitations.

### 4.2. Adsorption Separation Methods

Without affecting the efficacy of medicinal materials and other components, AAs are separated and enriched to eliminate toxicity. The reported methods for the separation and enrichment of AAs include solid-phase extraction (SPE), electromembrane extraction (EME), Quick, Easy, Cheap, Effective, Rugged, and Safe (QuEChERS), and supercritical fluid extraction (SFE).

#### 4.2.1. SPE

SPE plays an important role in sample pretreatment in biological, food, and environmental analyses. The whole operation process is divided into four parts: pretreatment, loading, washing, desorption, and collection. SPE offers the advantages of a low cost, simple operation, and high recovery rate. As the core of SPE, the solid-phase extractant functions in the recovery, reproducibility, and accuracy of analytes. SPE materials for the separation and purification of AAs include MOFs, carbon nanotubes (CNTs), carbon microcoils (CMCs), and molecularly imprinted polymers (MIPs).

(1) MOFs

MOFs comprise a hybrid network structure material with organic and inorganic properties. Given their ultrahigh specific surface area and controllable structure, MOFs have attracted the attention of researchers investigating SPE. To enhance the adsorption capacity of MOFs, Fang et al. [88] used an ionic liquid to modify the surface of the MOFs material ZIF-67 with ionic liquid. The adsorbent reached an adsorption capacity of 34.25 mg/g within 138 min, and AAI was separated from natural plants. Zhang et al. [89] modified UiO-66-NH_2_ and combined it with N-methylacrylamide (NMA). The obtained material was used for the online enrichment and purification of AAI in medicinal plants. The main interactions in the adsorption process include hydrogen bonding, electrostatic interaction, and π stacking [90]. The combination of melamine and MOFs can not only increase the specific surface area but also enhance the interaction between groups and improve the adsorption performance. Shu et al. [91] applied such a method to enrich and detect AAI in traditional Chinese medicine. Cheng et al. [92] used MIL-101 (Fe) to adsorb AAI and AAII in Houttuynia cordata and achieved a good adsorption effect. As an adsorption material, the defect of MOF materials depends on their instability in water, in which heavy metal ions easily leak and cause secondary pollution to the test sample.

(2) CNTs

CNTs can be considered a layer of graphene curled into nano-sized tubes. They can be combined with organic molecules through covalent or noncovalent bonds for the selective adsorption of analytes. To separate and purify AAs, Li et al. [93] used the sol–gel method to synthesize magnetic CNTs (MCNTs) and coated their surface with MIP (AAI-MIPs) film. The prepared MCNTs @AAI-MIPs presented a fast separation speed and a large adsorption capacity for AAI in traditional Chinese medicine samples. Similarly, Shu et al. [94] coated MCNTs with adenine. Their experiment showed that the adenine-functionalized adsorbent attained an adsorption capacity for AAI equal to 24.5 µg/mg.

(3) CMCs

CMCs have attracted considerable attention due to their helical morphology, super-elasticity, electromagnetic properties, hydrogen absorption, and thermal stability [95]. Liu et al. [96] used Cucurbitaceae plants as precursors to prepare spiral CMCs. Following Liu’s idea regarding the preparation of CMCs, Shu et al. [97] compounded such materials with chitosan to synthesize CMCs@CS for the selective separation of AAI from medicinal materials. The experimental results showed the excellent adsorption capacity of CMCs@CS (77.72 mg/g).

(4) MIPs

**Figure 3:**
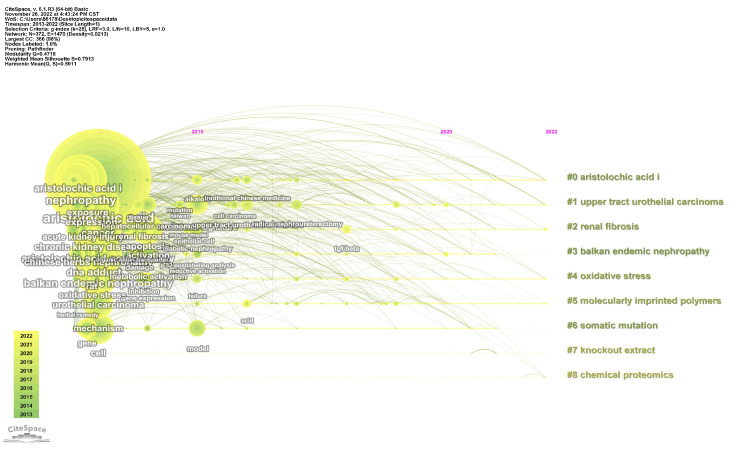
Cluster analysis of keywords from the literature related to AAs from 2013 to 2022. Produced using CiteSpace 6.1. R3, Excel, and Origin 2019.

**Figure 4:**
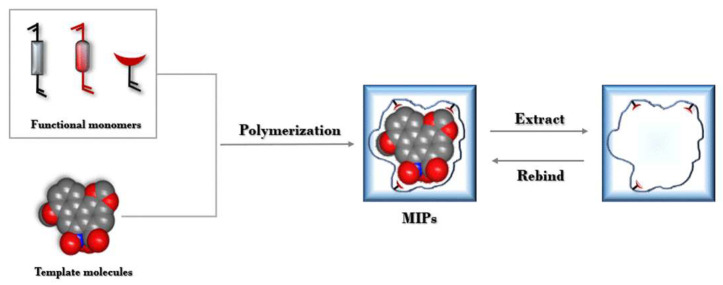
The steps of the molecular imprinting technique (using AAs as template molecules).

Using the core collection database of the Science Network as the data source, this paper analyzed the literature related to AAs from 2013 to 2022. CiteSpace 6.1. The R3 analysis tool was employed to cluster keywords in the text. Cluster analysis was conducted to group data into three categories, namely headings, keywords, or abstracts, to visualize the current research state in the field of AAs. Figure 3 presents the cluster analysis of the literature on AA keywords from 2013 to 2022, where Q = 0.4718 and S = 0.7913, which indicate relatively clear clustering results. The graph shows eight large clusters, with MIPs gaining increasing interest over the last decade. The use of MIPs is inspired by the specific recognition of natural antibody–antigen binding (Figure 4). The procedure involves three stages: (1) the combination of target molecules with template molecules to create MIPs in a covalent or noncovalent form; (2) the removal of target molecules from the polymer, with specific cavities left behind; and (3) the adsorption of target molecules from complex samples by the cavities for adsorption separation [98]. 

The development of extraction materials with favorable properties for enriching and separating target substances from complex systems is crucial for solid-phase extraction. MIPs play a central role in the molecular imprinting process. Various methods have been employed to prepare MIPs, including native polymerization [99], in situ polymerization [100], precipitation polymerization [101], suspension polymerization, and sol–gel methods. For instance, Xiao et al. [102] synthesized MIPs using AA I as the template molecule through the reversible addition-rupture chain transfer process precipitation polymerization method. Li et al. [93] used a simple sol–gel method to self-assemble the template molecule AA I with functional monomers, synthesizing magnetic carbon nanotubes functionalized with MIPs. Zhang et al. [89] developed composite monolithic columns with metal–organic frameworks through in situ polymerization, allowing for the purification and enrichment of AA I from medicinal plants. As researchers strive for greater morphology and functionality from MIPs, increased adsorption capacities and rates are necessary. For example, Xiong et al. [103] synthesized magnetic spherical thermal MIPs capable of thermally capturing/releasing AA I, enabling rapid magnetic separation, shorter elution times, and reduced organic solvent consumption. Shu et al. [91] prepared octahedral-shaped MIPs using melamine as the recognition unit and grafted it onto the surface of a metal–organic framework for the effective and exclusive adsorption of AA I. However, some methods may have drawbacks, such as toxicity, high cost, instability, or the leakage of template molecules. Ge et al. [104] used indomethacin molecules as virtual template molecules to prepare magnetic mesoporous carbon MIPs for the selective recognition of AA I and AA II from rat urine samples, offering a potential solution to these challenges. Moreover, dummy template imprinting has been explored for the selective separation of AAI in Chinese medicine, showing good recovery and precision values [105]. Wang et al. [106] prepared molecularly imprinted silica materials using the virtual template molecule 1,10-phenanthroline-4-carboxylic acid, which exhibited the strong adsorption of AAI in root extracts of natural plants, reaching 61.3% of the maximum adsorption capacity within 1 min. Various factors influence the preparation of molecularly imprinted polymers, including template molecules, functional monomers, crosslinkers, solvents, and polymerization temperature, among others. Table 2 provides a summary of the current preparation stages of MIPs for AAs.

#### 4.2.2. EME

EME is a hybrid technology integrating electrophoresis and separation. The method is implemented under the following working principle: under the action of an electric field, the charged analyte in the sample migrates to the analytical solution through a supported liquid film (SLM) and is enriched in the analytical solution, which results in sample purification [107]. For the first time, Yan et al. [108] used EME to extract AAI and AAII from urine samples. The linear range was 10–1000 ng/mL (R^2^ ≥ 0.9970), and the detection limits were 2.7 and 2.9 ng/mL, respectively.

#### 4.2.3. QuEChERS Method

QuEChERS is a dispersive SPE sample preparation technique that uses adsorbents to adsorb impurities for purification [109]. In the work of Zhang et al. [110], this method was applied to plant and soil samples and Chinese patent medicine samples. The plant and soil samples were pretreated through extraction and high-speed centrifugation. Lukas et al. [61] studied the extraction of AAI and AAII in various herbal dietary supplements based on the QuEChERS method. Compared with SPE, the improved QuEChERS method has a lower cost, consumes less time, and has a lower labor intensity in sample purification.

#### 4.2.4. SFE

SFE uses the influence of pressure and temperature on the solubility of supercritical fluids. In the supercritical state, the supercritical fluid contacts with the substance to be separated and selectively extracts the components with a certain molecular weight, polarity, and boiling point [111]. Liang et al. [112] used SFE to extract AAI and AAII from plants under prerequisite conditions that exceed the critical temperature and pressure.

### 4.3. Chemical Methods

**Figure 5:**
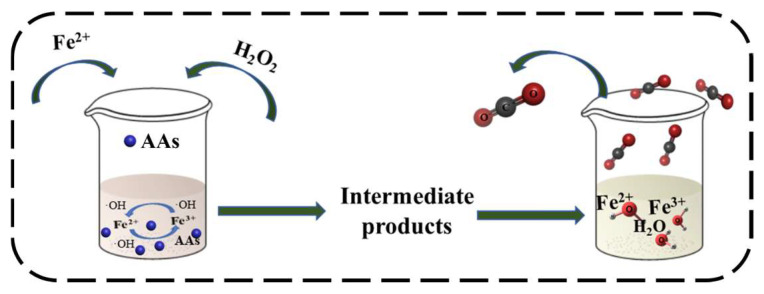
The process of removing AAs by the Fenton method.

Microbial and physical methods are commonly used to purify and detoxify AAs in drugs. However, the impact of AAs on the environment also needs to be addressed. Chan et al. [113] used advanced oxidation technology to investigate the degradation of AAs in contaminated soil and water. By exploiting the interconversion of Fe^2+^ and Fe^3+^ during the reaction, they utilized H_2_O_2_ to produce hydroxyl radicals and determined the optimal reaction conditions. The degradation effect achieved an efficiency of 97% at a soil concentration of 500 µg/kg. This literature formed the basis for future research utilizing Fenton technology for AAs degradation in the environment. The Fenton technique enables the complete oxidation and decomposition of complex organic molecules into CO_2_ and H_2_O (Figure 5).

**Table 2:** Current methods of making MIPs.

Template Molecules	Functional Monomers	Crosslinkers	Initiators	MIPs	Recovery (%)	RSDs (%)	LOD (µg/L)	Adsorption Capacity (mg/g)	Ref
AAI	ZIF-67	NBS	AIBN	ZIF-67 @EIM-MIM	96.2–100.0%	3.5–4.0%	20	50.9	[114]
AAI	acrylic acid	EGDMA	AIBN	MIPs	91.5%	<4.2%	60	1.3	[102]
AAI	SiO_2_	TRIM	—	IL-IL @silicas	70.0–110.6%	3.5–9.1%	—	16.7	[115]
AAI, AAII	CNT	GPTMS	—	SiO_2_@CNT/Fe_3_O_4_	92.7–97.5% 92.6–99.4%	<4.0%	50	24.5	[94]
AAI	Chitosan	—	—	CMCs @CS	73.6–77.7%	0.8–4.5%	10	77.72	[97]
AAI	PTMOS	TEOS	HAc	MCNTs @AAI-MIPs	80–110%	3.27–8.16%	34	23.96	[93]
AAI	ZIF-67	EGDMA	AIBN	GEIM-ZIF-67	97.67–106.98%	—	24.1	34.25	[88]

Notes: Carbon nanotubes (CNT), phenyltrimethoxysilane (PTMOS), N-bromosuccinimide (NBS), ethylene dimethacrylate (EGDMA), (3-chloropropyl) trimethoxysilane (TRIM), trimethoxysilane (GPTMS), silicic acid (TEOS), azodiisobutyronitrile (AIBN), acetic acid (HAc).

## 5. Conclusions

This article aimed to improve people’s comprehension of AAs in the environment by reviewing the related carcinogenic properties and exposure pathways. This work emphasized the necessity of studying AA degradation in the environment. The review of the detection methods for AAs revealed the strong instrument dependence of HPLC, HPLC-MS, the fluorescence sensor method, and near-infrared spectroscopy. Biological detection methods have certain advantages, such as a shorter detection time and lower cost, over the above methods. The sensitivity of biological detection methods for the detection of pollutants needs to be improved. For the removal of AAs, the microbial method has great advantages in the detoxification of drugs and the environment. It can carry out a wide range of in situ repairs and reduce the sampling cost. The physical adsorption method can be applied to purify drugs without affecting their efficacy. However, the later elution problem, which is needed to enhance the stability of the adsorbent material, must be considered. For chemical treatment, this paper proposes the Fenton technology in advanced oxidation technology as an effective method for the analysis of the intermediate products formed during degradation and the evaluation of their toxicity. For minimized secondary pollution, chemical bond breaking in the structure of AAs provides the possibility of solving the problem related to these harmful pollutants from the environmental source.

## Figures and Tables

**Table 1:** Summary of relevant detection methods.

Analysis Method	Sample	Mass Spectrometer Type	Detection Pattern	Components	Scan Mode	Ref
LC-MS/MS	serum albumin	triple quadrupole	positive ion mode	protein adducts of AAI	multiple reaction monitoring (MRM)	[55]
LC-MS/MS	herbal	quadrupole ion-trap	positive or negative ion mode	AAI, AAII	—	[56]
LC-MS/MS	Chinese medicine	—	positive ion mode	AAI, AAII	full scan (SCAN)	[57]
LC-MS/MS	plasma metabolome	quadrupole time-of-flight	positive or negative ion mode	AAI, AAII	—	[58]
LC-MS/MS	rat plasma	triple quadrupole	positive ion mode	AAI	multiple reaction monitoring (MRM)	[59]
UHPLC-MS^n^	Human Tissues	quadrupole ion-trap	—	Aristolactam-DNA Adducts	multiple reaction monitoring (MRM)	[60]
UHPLC-MS^3^	herbal	quadrupole ion-trap	positive ion mode	AAI, AAII	multiple reaction monitoring (MRM)	[61]
LC-MS/MS	food and agricultural soil	—	positive ion mode	Aristolactams	multiple reaction monitoring (MRM)	[62]
LC-MS/MS	over 130 soil samples	—	positive ion mode	AAI	multiple reaction monitoring (MRM)	[63]
LC-MS/MS	Chinese patent medicine	triple quadrupole	positive ion mode	Aristolochic acid analogues	multiple reaction monitoring (MRM)	[64]
LC-MS/MS	Houttuynia cordata	—	positive ion mode	AAI, AAII	multiple reaction monitoring (MRM)	[65]
UHPLC-MS	Houttuynia cordata	quadrupole time-of-flight	—	Aristolochic acid analogues	—	[66]
UHPLC-MS/MS	Aristolochiaceae plants	—	positive ion mode	Aristolochic acids analogs	dynamic multiple reaction monitoring (dMRM)	[67]
UHPLC-MS	Asari Radix et Rhizoma	triple quadrupole	positive ion mode	Aristolochic acids derivatives	multiple reaction monitoring (MRM)	[68]

## Data Availability

The data presented in this study will be available on request.

## References

[1] Kiliś-Pstrusińska K., Wiela-Hojeńska A. (2021). Nephrotoxicity of Herbal Products in Europe-A Review of an Underestimated Problem. Int. J. Mol. Sci..

[2] Uber A.M., Sutherland S.M. (2020). Nephrotoxins and nephrotoxic acute kidney injury. Pediatr. Nephrol..

[3] Debelle F.D., Vanherweghem J.L., Nortier J.L. (2008). Aristolochic acid nephropathy: A worldwide problem. Kidney Int..

[4] Heinrich M., Chan J., Wanke S., Neinhuis C., Simmonds M.S.J. (2009). Local uses of Aristolochia species and content of nephrotoxic aristolochic acid 1 and 2-A global assessment based on bibliographic sources. J. Ethnopharmacol..

[5] Vanherweghem J.L., Depierreux M., Tielemans C., Abramowicz D., Dratwa M., Jadoul M., Richard C., Vandervelde D., Verbeelen D., Vanhaelenfastre R. (1993). Rapidly progressive interstitial renal fibrosis in young-women—Association with slimming regimen including chinese herbs. Lancet.

[6] De Broe M.E. (1999). On a nephratoxic and carcinogenic slimming regimen. Am. J. Kidney Dis..

[7] Schmeiser H.H., Bieler C.A., Wiessler M., Strihou C.v.Y.d., Cosyns J.-P. (1996). Detection of DNA Adducts Formed by Aristolochic Acid in Renal Tissue from Patients with Chinese Herbs Nephropathy. Cancer Res..

[8] Wolf G., Porth J., Stahl R.A. (2001). Thrombosis associated with cytomegalovirus infection in patients with ANCA-positive vasculitis. Am. J. Kidney Dis..

[9] Thangavelu M., Ismail A., Zakaria A., Elmansy H., Shahrour W., Prowse O., Kotb A. (2022). Aristolochic acid: What urologists should know. Arch. Ital. Urol. Androl..

[10] Han J.Y., Xian Z., Zhang Y.S., Liu J., Liang A.H. (2019). Systematic Overview of Aristolochic Acids: Nephrotoxicity, Carcinogenicity, and Underlying Mechanisms. Front. Pharmacol..

[11] Abdullah R., Alhusainy W., Woutersen J., Rietjens I.M., Punt A. (2016). Predicting points of departure for risk assessment based on in vitro cytotoxicity data and physiologically based kinetic (PBK) modeling: The case of kidney toxicity induced by aristolochic acid I. Food Chem. Toxicol..

[12] Poon S.L., Pang S.T., McPherson J.R., Yu W., Huang K.K., Guan P., Weng W.H., Siew E.Y., Liu Y., Heng H.L. (2013). Genome-Wide Mutational Signatures of Aristolochic Acid and Its Application as a Screening Tool. Sci. Transl. Med..

[13] Grosse Y., Baan R., Straif K., Secretan B., El Ghissassi F., Bouvard V., Benbrahim-Tallaa L., Guha N., Galichet L., Cogliano V. (2009). A review of human carcinogens—Part A: Pharmaceuticals. Lancet Oncol..

[14] Ng A.W.T., Poon S.L., Huang M.N., Lim J.Q., Boot A., Yu W., Suzuki Y., Thangaraju S., Ng C.C.Y., Tan P. (2017). Aristolochic acids and their derivatives are widely implicated in liver cancers in Taiwan and throughout Asia. Sci. Transl. Med..

[15] Lu Z.N., Luo Q., Zhao L.N., Shi Y., Wang N., Wang L., Han Z.G. (2020). The Mutational Features of Aristolochic Acid-Induced Mouse and Human Liver Cancers. Hepatology.

[16] Fang Z.E., Wang C.Y., Niu M., Liu T.T., Ren L.T., Li Q., Li Z.Y., Wei Z.Y., Lin L., Mu W.Q. (2022). Integration of Transcriptomic and Metabolomic Data to Compare the Hepatotoxicity of Neonatal and Adult Mice Exposed to Aristolochic AcidⅠ. Front. Genet..

[17] Bamias G., Boletis J. (2008). Balkan nephropathy: Evolution of our knowledge. Am. J. Kidney Dis..

[18] Petrescu A.M., Lukinich-Gruia A.T., Paunescu V., Ilia G. (2019). A Theoretical Study of the Molecular Coupled Structures of Aristolochic Acids and Humic Acid, Potential Environmental Contaminants. Chem. Biodivers..

[19] Gokmen M.R., Cosyns J.P., Arlt V.M., Stiborova M., Phillips D.H., Schmeiser H.H., Simmonds M.S.J., Cook H.T., Vanherweghem J.L., Nortier J.L. (2013). The Epidemiology, Diagnosis, and Management of Aristolochic Acid Nephropathy A Narrative Review. Ann. Intern. Med..

[20] Jelaković B., Dika Ž., Arlt V.M., Stiborova M., Pavlović N.M., Nikolić J., Colet J.M., Vanherweghem J.L., Nortier J.L. (2019). Balkan Endemic Nephropathy and the Causative Role of Aristolochic Acid. Semin. Nephrol..

[21] Kang Y.C., Chen M.H., Lin C.Y., Lin C.Y., Chen Y.T. (2021). Aristolochic acid-associated urinary tract cancers: An updated meta-analysis of risk and oncologic outcomes after surgery and systematic review of molecular alterations observed in human studies. Ther. Adv. Drug Saf..

[22] Anger E.E., Yu F., Li J. (2020). Aristolochic Acid-Induced Nephrotoxicity: Molecular Mechanisms and Potential Protective Approaches. Int. J. Mol. Sci..

[23] Nortier J.L., Vanherweghem J.-L., Jelakovic B. (2022). Aristolochic Acid Nephropathy and Balkan Nephropathy. Tubulointerstitial Nephritis.

[24] Schmeiser H.H., Stiborova M., Arlt V.M. (2009). Chemical and molecular basis of the carcinogenicity of Aristolochia plants. Curr. Opin. Drug Discov. Dev..

[25] Reddy M.V., Randerath K. (1986). Nudease Pl-mediated enhancement of sensitivity of 32P-postlabeling test for structurally diverse DNA adducts. Carcinogenesis.

[26] Pfau W., Schmeiser H.H., Wiessler M. (1990). AristolocMc acid binds covafleettly to tlhe exocycMc ammo gromp off perime emclleoitides mDNA. Carcinogenesis.

[27] Pfau W., Schmeiser H.H., Wiessler M. (1990). 32P-PostIlaIbeIlMinig amalysns off tihe DNA addmdts ffonnnied by aristoIocMc acid I and II. Carcinogenesis.

[28] Attaluri S., Bonala R.R., Yang I.Y., Lukin M.A., Wen Y.J., Grollman A.P., Moriya M., Iden C.R., Johnson F. (2010). DNA adducts of aristolochic acid II: Total synthesis and site-specific mutagenesis studies in mammalian cells. Nucleic Acids Res..

[29] Grollman A.P., Shibutani S., Moriya M., Miller F., Wu L., Moll U., Suzuki N., Fernandes A., Rosenquist T., Medverec Z. (2007). Aristolochic acid and the etiology of endemic (Balkan) nephropathy. Proc. Natl. Acad. Sci. USA.

[30] Arlt V.M., Stiborova M., Schmeiser H.H. (2002). Aristolochic acid as a probable human cancer hazard in herbal remedies: A review. Mutagenesis.

[31] Jadot I., Decleves A.E., Nortier J., Caron N. (2017). An Integrated View of Aristolochic Acid Nephropathy: Update of the Literature. Int. J. Mol. Sci..

[32] Xian Z., Tian J.Z., Zhang Y.S., Meng J., Zhao Y., Li C.Y., Yi Y., Han J.Y., Liu S.Y., Wang L.M. (2021). Study on the potential nephrotoxicity and mutagenicity of aristolochic acid IVa and its mechanism. Biomed. Pharmacother..

[33] Das S., Thakur S., Korenjak M., Sidorenko V.S., Chung F.F.L., Zavadil J. (2022). Aristolochic acid-associated cancers: A public health risk in need of global action. Nat. Rev. Cancer.

[34] Voice T.C., McElMurry S.P., Long D.T., Dimitrov P., Ganev V.S., Petropoulos E.A. (2006). Evaluation of the hypothesis that Balkan endemic nephropathy is caused by drinking water exposure to contaminants leaching from Pliocene coal deposits. J. Expo. Sci. Environ. Epidemiol..

[35] Li W.W., Hu Q., Chan W. (2016). Uptake and Accumulation of Nephrotoxic and Carcinogenic Aristolochic Acids in Food Crops Grown in *Aristolochia clematitis*-Contaminated Soil and Water. J. Agric. Food Chem..

[36] Li W.W., Chan C.K., Liu Y.S., Yao J., Mitic B., Kostic E.N., Milosavljevic B., Davinic I., Orem W.H., Tatu C.A. (2018). Aristolochic Acids as Persistent Soil Pollutants: Determination of Risk for Human Exposure and Nephropathy from Plant Uptake. J. Agric. Food Chem..

[37] Chan W., Pavlovic N.M., Li W.W., Chan C.K., Liu J.J., Deng K.L., Wang Y.N., Milosavljevic B., Kostic E.N. (2016). Quantitation of Aristolochic Acids in Corn, Wheat Grain, and Soil Samples Collected in Serbia: Identifying a Novel Exposure Pathway in the Etiology of Balkan Endemic Nephropathy. J. Agric. Food Chem..

[38] Pavlovic N.M., Maksimovic V., Maksimovic J.D., Orem W.H., Tatu C.A., Lerch H.E., Bunnell J.E., Kostic E.N., Szilagyi D.N., Paunescu V. (2013). Possible health impacts of naturally occurring uptake of aristolochic acids by maize and cucumber roots: Links to the etiology of endemic (Balkan) nephropathy. Environ. Geochem. Health.

[39] Chan C.-K., Xiong L., Pavlović N.M., Chan W. (2021). Determination of Aristolochic Acids in Soil, Water, and Herbal Plants in Medicinal Plant Cultivation Areas: An Emerging Environmental Contaminant Worth Concerning. ACS Agric. Sci. Technol..

[40] Zhang J., Wang Y., Wang C., Li K., Tang W., Sun J., Wang X. (2022). Uptake, Translocation, and Fate of Carcinogenic Aristolochic Acid in Typical Vegetables in Soil-Plant Systems. Molecules.

[41] Maharaj S.V.M., Orem W.H., Tatu C.A., Lerch H.E., Szilagyi D.N. (2014). Organic compounds in water extracts of coal: Links to Balkan endemic nephropathy. Environ. Geochem. Health.

[42] Tung K.K., Chan C.K., Zhao Y., Chan K.K.J., Liu G.R., Pavlovic N.M., Chan W. (2020). Occurrence and Environmental Stability of Aristolochic Acids in Groundwater Collected from Serbia: Links to Human Exposure and Balkan Endemic Nephropathy. Environ. Sci. Technol..

[43] Lukinich-Gruia A.T., Nortier J., Pavlovic N.M., Milovanovic D., Popovic M., Draghia L.P., Paunescu V., Tatu C.A. (2022). Aristolochic acid I as an emerging biogenic contaminant involved in chronic kidney diseases: A comprehensive review on exposure pathways, environmental health issues and future challenges. Chemosphere.

[44] Lee T.-Y., Wu M.-L., Deng J.-F., Hwang D.-F. (2001). High-performance liquid chromatographic determination for aristolochic acid in medicinal plants and slimming products. J. Chromatogr. B.

[45] Kotani A., Kotani T., Kojima S., Hakamata H., Kusu F. (2014). Determination of Aristolochic Acids I and II in Herbal Medicines by High-performance Liquid Chromatography with Electrochemical Detection. Electrochemistry.

[46] Chan W., Lee K.C., Liu N., Cai Z.W. (2007). A sensitivity enhanced high-performance liquid chromatography fluorescence method for the detection of nephrotoxic and carcinogenic aristolochic acid in herbal medicines. J. Chromatogr. A.

[47] Yue H., Chan W., Guo L., Cai Z. (2009). Determination of aristolochic acid I in rat urine and plasma by high-performance liquid chromatography with fluorescence detection. J. Chromatogr. B Anal. Technol. Biomed. Life Sci..

[48] Wang Y.A., Chan W. (2014). Determination of Aristolochic Acids by High-Performance Liquid Chromatography with Fluorescence Detection. J. Agric. Food Chem..

[49] Li W.W., Chan C.K., Wong Y.L., Chan K.K.J., Chan H.W., Chan W. (2018). Cooking methods employing natural anti-oxidant food additives effectively reduced concentration of nephrotoxic and carcinogenic aristolochic acids in contaminated food grains. Food Chem..

[50] Chin M.L., Au C.K., Chan C.K., Jin L., Zivkovic Stosic M.Z., Dordevic Zlatkovic M.R., Zlatkovic D., Pavlovic N.M., Chan W. (2023). Fabrication of a Simple and Efficient HPLC Reduction Column for Online Conversion of Aristolochic Acids to Aristolactams Prior to Sensitive Fluorescence Detection. Anal. Chem..

[51] Yuan F., Zhang D.W., Liu J.X., Zhou Y.L., Zhang X.X. (2016). Phytochemical profiling in single plant cell by high performance liquid chromatography-mass spectrometry. Analyst.

[52] Chan W., Zheng Y.F., Cai Z.W. (2007). Liquid chromatography-tandem mass spectrometry analysis of the DNA adducts of aristolochic acids. J. Am. Soc. Mass Spectrom..

[53] Leung E.M., Chan W. (2015). Quantification of aristolochic acid-RNA adducts in the urine of aristolochic acid-treated rats by liquid chromatography-tandem mass spectrometry. Chem. Res. Toxicol..

[54] Draghia L.P., Lukinich-Gruia A.T., Oprean C., Pavlovic N.M., Paunescu V., Tatu C.A. (2021). Aristolochic acid I: An investigation into the role of food crops contamination, as a potential natural exposure pathway. Environ. Geochem. Health.

[55] Chan C.K., Chan K.K.J., Liu N., Chan W. (2021). Quantitation of Protein Adducts of Aristolochic Acid I by Liquid Chromatography-Tandem Mass Spectrometry: A Novel Method for Biomonitoring Aristolochic Acid Exposure. Chem. Res. Toxicol..

[56] Kite G.C., Yule M.A., Leon C., Simmonds M.S.J. (2002). Detecting aristolochic acids in herbal remedies by liquid chromatography/serial mass spectrometry. Rapid Commun. Mass Spectrom..

[57] Jong T.T., Lee M.R., Hsiao S.S., Hsai J.L., Wu T.S., Chiang S.T., Cai S.Q. (2003). Analysis of aristolochic acid in nine sources of Xixin, a traditional Chinese medicine, by liquid chromatography/atmospheric pressure chemical ionization/tandem mass spectrometry. J. Pharm. Biomed. Anal..

[58] Lin S.H., Chan W., Li J.H., Cai Z.W. (2010). Liquid chromatography/mass spectrometry for investigating the biochemical effects induced by aristolochic acid in rats: The plasma metabolome. Rapid Commun. Mass Spectrom..

[59] Liu Y.M., Lin A.H., Wu Z.F., Ou R.M., Huang H.D. (2010). A liquid chromatography/tandem mass spectrometry method for determination of aristolochic acid-I in rat plasma. Biomed. Chromatogr..

[60] Yun B.H., Rosenquist T.A., Sidorenko V., Iden C.R., Chen C.H., Pu Y.S., Bonala R., Johnson F., Dickman K.G., Grollman A.P. (2012). Biomonitoring of Aristolactam-DNA Adducts in Human Tissues Using Ultra-Performance Liquid Chromatography/Ion-Trap Mass Spectrometry. Chem. Res. Toxicol..

[61] Vaclavik L., Krynitsky A.J., Rader J.I. (2014). Quantification of aristolochic acids I and II in herbal dietary supplements by ultra-high-performance liquid chromatography-multistage fragmentation mass spectrometry. Food Addit. Contam. Part A—Chem. Anal. Control Expo. Risk Assess..

[62] Chan C.K., Pavlovic N.M., Chan W. (2019). Development of a novel liquid chromatography-tandem mass spectrometric method for aristolochic acids detection: Application in food and agricultural soil analyses. Food Chem..

[63] Chan C.K., Chan K.K.J., Pavlovic N.M., Chan W. (2020). Liquid chromatography-tandem mass spectrometry analysis of aristolochic acids in soil samples collected from Serbia: Link to Balkan endemic nephropathy. Rapid Commun. Mass Spectrom..

[64] Liu J., Liu Y., Wu Y.X., Dai Z., Ma S.C. (2020). Rapid Analysis of Aristolochic Acid Analogues in Traditional Chinese Patent Medicine by LC-MS/MS. J. Anal. Methods Chem..

[65] Chan C.K., Pan G.R., Chan W. (2021). Analysis of aristolochic acids in *Houttuynia cordataby* liquid chromatography-tandem mass spectrometry. J. Mass Spectrom..

[66] Yu X., Gao Y., Xu Y., Guo X., Guo L., Tan T., Liu F., Wan Y.Q. (2022). Study of the Contents of Analogues of Aristolochic Acid in *Houttuynia cordata* by Ultra-High Performance Liquid Chromatography Tandem Mass Spectrometry. Foods.

[67] Ji H., Zhang G., Zhou X. (2023). Rapid simultaneous determination of thirteen aristolochic acids analogs in *Aristolochiaceae* plants by Ultra-High-Performance liquid Chromatography-tandem mass spectrometry in dynamic multiple reaction monitoring mode. J. Chromatogr. B Anal. Technol. Biomed. Life Sci..

[68] Liu H., Cheng X., Guan H., Wang C. (2022). Rapid and Simultaneous Quantification of Six Aristolochic Acids and Two Lignans in Asari Radix et Rhizoma Using Ultra-Performance Liquid Chromatography-Triple Quadrupole Tandem Mass Spectrometry. J. Anal. Methods Chem..

[69] Liu J.L., Xu C.L., Yang T., Hu Z.R., Zhang Z.Q., Feng G.D. (2019). Developed a novel sensor based on fluorescent graft conjugated polymer for the determination of aristolochic acid in traditional Chinese medicine. Spectrochim. Acta Part A—Mol. Biomol. Spectrosc..

[70] Song L.J., Liu M.Y., Tian F.L., Liu Z.L. (2021). A Novel Luminescent Metal-Organic Framework as a Remarkable Sensor for Detecting Aristolochic Acids in Biological Fluids. Eur. J. Inorg. Chem..

[71] Shen X., Yan B. (2023). Europium chelate-anionic exchange functionalized covalent organic frameworks for the sensing of aristolochic acid a in humans and sulfamethoxazole/trimethoprim in surface water. Talanta.

[72] Li W., Gong S., Wen D., Che B., Liao Y., Liu H., Feng X., Hu S. (2004). Rapid determination of aristolochic acid I and II in Aristolochia plants from different regions by β-cyclodextrin-modified capillary zone electrophoresis. J. Chromatogr. A.

[73] Wang Y., Mamat X., Li Y., Hu X., Wang P., Dong Y., Hu G. (2019). Glassy Carbon Electrode Modified via Molybdenum Disulfide Decorated Multiwalled Carbon Nanotubes for Sensitive Voltammetric Detection of Aristolochic Acids. Electroanalysis.

[74] Wang Y., Qiao M.F., Baikeli Y., Mamat X., Li L., Hu X., Dong Y.M., Chang F.Q., Zhang H.C., Hu G.Z. (2020). Soft-templated mesoporous carbon-modified glassy carbon electrode for sensitive and selective detection of aristolochic acids. J. Hazard. Mater..

[75] Yang Z., Han L., Wang C., Li J., Fernández Pierna J.A., Dardenne P., Baeten V. (2016). Detection of Melamine in Soybean Meal Using Near-Infrared Microscopy Imaging with Pure Component Spectra as the Evaluation Criteria. J. Spectrosc..

[76] Chen X.Y., Chai Q.Q., Lin N., Li X.H., Wang W. (2019). 1D convolutional neural network for the discrimination of aristolochic acids and their analogues based on near-infrared spectroscopy. Anal. Methods.

[77] Wong Y.L., Wong K.L., Shaw P.C. (2015). Rapid authentication of Cordyceps by lateral flow dipstick. J. Pharm. Biomed. Anal..

[78] Chen S., Pang X., Song J., Shi L., Yao H., Han J., Leon C. (2014). A renaissance in herbal medicine identification: From morphology to DNA. Biotechnol. Adv..

[79] Wu L., Sun W., Wang B., Zhao H.Y., Li Y.L., Cai S.Q., Xiang L., Zhu Y.J., Yao H., Song J.Y. (2015). An integrated system for identifying the hidden assassins in traditional medicines containing aristolochic acids. Sci. Rep..

[80] Xin T., Xu Z., Jia J., Leon C., Hu S., Lin Y., Ragupathy S., Song J., Newmaster S.G. (2018). Biomonitoring for traditional herbal medicinal products using DNA metabarcoding and single molecule, real-time sequencing. Acta Pharm. Sin. B.

[81] Thongkhao K., Tungphatthong C., Sukrong S. (2022). A PCR-lateral flow immunochromatographic assay (PCR-LFA) for detecting Aristolochia species, the plants responsible for aristolochic acid nephropathy. Sci. Rep..

[82] Chen X., Zhang J., Xie J., Huang Z. (2023). Development of two immunochromatographic test strips based on gold nanospheres and gold nanoflowers for the rapid and simultaneous detection of aflatoxin B1 and aristolochic acid a in dual-use medicinal and food ingredients. Microchem. J..

[83] Ai S., Tang W., Guo R.L., Li J.Q., Yang W., He Z.G. (2019). Research progress on Chinese herbal medicine fermentation and profile of active substances derived. Zhongguo Zhong Yao Za Zhi.

[84] Sun Y., Ren G., Shi Q., Zhu H., Zhou N., Kong X., Jiang D., Liu C. (2023). Identification of a Novel Coumarins Biosynthetic Pathway in the Endophytic Fungus Fusarium oxysporum GU-7 with Antioxidant Activity. Appl. Environ. Microbiol..

[85] Zikmundova M., Drandarov K., Bigler L., Hesse M., Werner C. (2002). Biotransformation of 2-benzoxazolinone and 2-hydroxy-1,4-benzoxazin-3-one by endophytic fungi isolated from Aphelandra tetragona. Appl. Environ. Microbiol..

[86] Melo R., Sanhueza L., Mendoza L., Cotoras M. (2019). Characterization of the fungitoxic activity on Botrytis cinerea of the aristolochic acids I and II. Lett. Appl. Microbiol..

[87] Wang X., Jiang D., Shi Q., Ren G., Liu C. (2022). Microbial degradation of aristolochic acid I by endophytic fungus A.h-Fs-1 of Asarum heterotropoides. Front. Microbiol..

[88] Fang L.W., Tian M.L., Row K.H., Yan X.M., Xiao W. (2019). Isolation of aristolochic acid I from herbal plant using molecular imprinted polymer composited ionic liquid-based zeolitic imidazolate framework-67. J. Sep. Sci..

[89] Zhang M.M., Liu H.Y., Han Y.M., Bai L.G., Yan H.Y. (2020). On-line enrichment and determination of aristolochic acid in medicinal plants using a MOF-based composite monolith as adsorbent. J. Chromatogr. B—Anal. Technol. Biomed. Life Sci..

[90] Xu Q., Zhou Q., Pan M.M., Dai L.C. (2020). Interaction between chlortetracycline and calcium-rich biochar: Enhanced removal by adsorption coupled with flocculation. Chem. Eng. J..

[91] Shu H., Chen G.N., Wang L., Cui X., Luo Z.M., Jing W.H., Chang C., Zeng A.G., Zhang J., Fu Q. (2021). Metal-organic framework grafted with melamine for the selective recognition and miniaturized solid phase extraction of aristolochic acid I from traditional Chinese medicine. J. Chromatogr. A.

[92] Cheng R., Mao X., Yu J., Liu F., Guo L., Luo D., Wan Y. (2023). A dispersive solid-phase extraction method for the determination of Aristolochic acids in *Houttuynia cordata* based on MIL-101(Fe): An analytes-oriented adsorbent selection design. Food Chem..

[93] Li F., Gao J., Li X.X., Li Y.J., He X.W., Chen L.X., Zhang Y.K. (2019). Preparation of magnetic molecularly imprinted polymers functionalized carbon nanotubes for highly selective removal of aristolochic acid. J. Chromatogr. A.

[94] Shu H., Chen G.N., Wang L., Cui X., Wang Q., Li W., Chang C., Guo Q., Luo Z.M., Fu Q. (2020). Adenine-coated magnetic multiwalled carbon nanotubes for the selective extraction of aristolochic acids based on multiple interactions. J. Chromatogr. A.

[95] Raghubanshi H., Dikio E.D., Naidoo E.B. (2016). The properties and applications of helical carbon fibers and related materials: A review. J. Ind. Eng. Chem..

[96] Liu R.L., Yu P., Luo Z.M., Bai X.F., Li X.Q., Fu Q. (2017). Single-helix carbon microcoils prepared via Fe(III)-osmotically induced shape transformation of zucchini (*Cucurbita pepo* L.) for enhanced adsorption and antibacterial activities. Chem. Eng. J..

[97] Shu H., Ge Y.H., Xu X.Y., Guo P.Q., Luo Z.M., Du W., Chang C., Liu R.L., Fu Q. (2018). Hybrid-type carbon microcoil-chitosan composite for selective extraction of aristolochic acid I from *Aristolochiaceae* medicinal plants. J. Chromatogr. A.

[98] BelBruno J.J. (2019). Molecularly Imprinted Polymers. Chem. Rev..

[99] Hasanah A.N., Soni D., Pratiwi R., Rahayu D., Megantara S., Mutakin (2020). Synthesis of Diazepam-Imprinted Polymers with Two Functional Monomers in Chloroform Using a Bulk Polymerization Method. J. Chem..

[100] Phungpanya C., Chaipuang A., Machan T., Watla-iad K., Thongpoon C., Suwantong O. (2018). Synthesis of prednisolone molecularly imprinted polymer nanoparticles by precipitation polymerization. Polym. Adv. Technol..

[101] Albu A.-M., Maior I., Nicolae C.A., Bocăneală F.L. (2016). Novel Pva Proton Conducting Membranes Doped with Polyaniline Generated by in-Situ Polymerization. Electrochim. Acta.

[102] Xiao Y.H., Xiao R., Tang J., Zhu Q.K., Li X.M., Xiong Y., Wu X.W. (2017). Preparation and adsorption properties of molecularly imprinted polymer via RAFT precipitation polymerization for selective removal of aristolochic acid I. Talanta.

[103] Xiong H.H., Fan Y., Mao X.J., Guo L., Yan A.P., Guo X., Wan Y.Q., Wan H. (2022). Thermosensitive and magnetic molecularly imprinted polymers for selective recognition and extraction of aristolochic acid. Food Chem..

[104] Ge Y.H., Shu H., Xu X.Y., Guo P.Q., Liu R.L., Luo Z.M., Chang C., Fu Q. (2019). Combined magnetic porous molecularly imprinted polymers and deep eutectic solvents for efficient and selective extraction of aristolochic acid I and II from rat urine. Mater. Sci. Eng. C—Mater. Biol. Appl..

[105] Ge Y.H., Guo P.Q., Xu X.Y., Chen G.N., Zhang X.M., Shu H., Zhang B.L., Luo Z.M., Chang C., Fu Q. (2017). Selective analysis of aristolochic acid I in herbal medicines by dummy molecularly imprinted solid-phase extraction and HPLC. J. Sep. Sci..

[106] Wang L.H., Zhang C.Y., Chen Y.J., Deng Q.L., Wang S. (2020). Dummy molecularly imprinted silica materials for effective removal of aristolochic acid I from kaempfer dutchmanspipe root extract. Microchem. J..

[107] Gjelstad A., Pedersen-Bjergaard S. (2014). Electromembrane extraction--three-phase electrophoresis for future preparative applications. Electrophoresis.

[108] Yan Y., Huang C., Shen X. (2019). Electromembrane extraction of aristolochic acids: New insights in separation of bioactive ingredients of traditional Chinese medicines. J. Chromatogr. A.

[109] Anastassiades M. (2003). Fast and Easy Multiresidue Method Employing Acetonitrile Extraction/Partitioning and “Dispersive Solid-Phase Extraction” for the Determination of Pesticide Residues in Produce. J. AOAC Int..

[110] Zhang J., Wang Y., Sun J., Zhou G., Jiang X., Wang X. (2020). Correction: QuEChERS pretreatment combined with high-performance liquid chromatography-tandem mass spectrometry for determination of aristolochic acids I and II in Chinese herbal patent medicines. RSC Adv..

[111] Pattiram P.D., Abas F., Suleiman N., Mohamad Azman E., Chong G.H. (2022). Edible oils as a co-extractant for the supercritical carbon dioxide extraction of flavonoids from propolis. PLoS ONE.

[112] Liang Q., Chow A.H.L., Wang Y., Tong H.H.Y., Zheng Y. (2010). Removal of toxic aristolochic acid components from *Aristolochia* plants by supercritical fluid extraction. Sep. Purif. Technol..

[113] Chan C.K., Tung K.K., Pavlovic N.M., Chan W. (2020). Remediation of aristolochic acid-contaminated soil by an effective advanced oxidation process. Sci. Total Environ..

[114] Chen P., Li X., Yan X., Tian M. (2021). Solid-Phase Extraction of Aristolochic Acid I from Natural Plant Using Dual Ionic Liquid-Immobilized ZIF-67 as Sorbent. Separations.

[115] Fang L.W., Tian M.L., Yan X.M., Xiao W., Row K.H. (2019). Dual ionic liquid-immobilized silicas for multi-phase extraction of aristolochic acid from plants and herbal medicines. J. Chromatogr. A.

